# Mandarin Biochar-TETA (MBT) prepared from *Citrus reticulata* peels for adsorption of Acid Yellow 11 dye from water

**DOI:** 10.1038/s41598-022-22359-x

**Published:** 2022-10-22

**Authors:** Ahmed Eleryan, Murat Yılmaz, Mohamed A. El-Nemr, Safaa Ragab, Mohamed Helal, Mohamed A. Hassaan, Ahmed El Nemr

**Affiliations:** 1 Environment Division, National Institute of Oceanography and Fisheries (NIOF), Kayet Bey, Elanfoushy, Alexandria, Egypt; 2 Department of Chemical Engineering, Faculty of Engineering, Osmaniye Korkut Ata University, 80000 Osmaniye, Turkey; 3 Department of Chemical Engineering, Faculty of Engineering, Minia University, Minia, Egypt

**Keywords:** Environmental sciences, Chemistry, Chemical engineering

## Abstract

Dehydration technique with 80% sulfuric acid was used to create a novel biochar from mandarin peel wastes followed by condensate with triethylenetetramine (TETA) to give Mandarin Biochar-TETA (MBT). BJH, BET, FTIR, SEM, DSC, TGA, and EDX studies were used to characterise the MBT. The capacity of the newly developed biochar to remove Acid Yellow 11 (AY11) dye from a water solution was studied. The pH of AY11 dye adsorption was found to be best at pH 1.5. Using 100 ppm AY11 dye as a beginning concentration and 1.75 g L^–1^ MBT dose, the greatest percent of AY11 dye removal by MBT was 97.83%. The MBT calculated maximum adsorption capacity (*Q*_m_) was 384.62 mg g^–1^. Langmuir (LIM), Freundlich (FIM), Tempkin (TIM), and Dubinin–Radushkevich (DRIM) isotherm models were applied to analyse the experimental data. Furthermore, the results of these isotherm models were investigated by various known error function equations. The MBT experimental data was best suited by the LIM. Pseudo-first-order (PFO), pseudo-second-order (PSO), Elovich kinetic model (EKM), intraparticle diffusion (IPD), and film diffusion (FD) models were used to calculate kinetic data. A PSO rate model with a high correlation (*R*^2^ > 0.990) was used to assess the adsorption rate. The main mechanism of the MBT adsorption method of the AY11 dye’s anions adsorption is the electrostatic attractive forces that arise with the increase of positively charged sites in an acidic medium. The obtained data suggest that the prepared MBT adsorbent has the potential to be an effective material to remove the AY11 dye from water and that it may be used repeatedly without losing its adsorption efficiency.

## Introduction

Concerns about water scarcity in sporadic places worldwide were growing significantly at this time, and this was exacerbated because of the continuing pollution of current water bodies in various locations^1^. Chemical contaminants that place a heavy burden on the ecosystem can be shown as dyes^2,3^, medicines^4^, heavy metals^5–7^, pesticides^8–11^ and hydrocarbons^12^. These contaminants are introduced into the water bodies via the discharge of industrial and hospital wastewater, as well as through home sewage systems. Because of their colors, dyes, in particular, can be easily distinguished in sewage samples. Synthetic dyes are the most often used dyes, and they are widely used in many industries, including textile, leather, paint, and other industries^2^. Because most colours are poisonous, carcinogenic, and non-biodegradable, they have a negative impact on the ecological balance as well as human health^13^. It is estimated that the amount of untreated dyes that are discharged in water bodies is between 0.7 and 2.0 × 10^5^ tonnes per year^14^, representing approximately 10–20% of total dyestuffs discharged. Inside artificial dyes produced annually, Azo dyes ranked first, because they have the largest number of colors and possess the largest wide range of uses. Overuse of these chemicals generates carcinogenic compounds^15^.

There are many methods to treat dyehouse effluent and the main ones can be classified as biological treatment^16^, electrochemical treatment^17^, chemical oxidation^18^, photo-degradation^19,20^, coagulation/flocculation^21^, Advanced oxidation^22,23^, adsorption treatment^24–26^. Because of its great efficiency, the adsorption technique using activated carbon (AC) for dye removal is one of the most often used among these techniques^27–29^. However, due to high production costs and the treatment of AC, scientists try to manufacture less expensive and more efficient adsorbent materials^30–36^. The trend toward the production of Biochar became a more cost-effective option and a clearer environment friendly. Biochar, which is derived from biomass and residues as raw materials, also prevents scarce resources from being wasted. Biochar is a term that refers to carbonaceous solids that are obtained through the pyrolysis of organic materials at a temperature greater than 300 °C in an environment of N_2_ gas^37–39^. Güzel et al.^40^ conducted an analysis and discovered that commercial AC manufacturing activities are more expensive than biochar production, according to their findings. Besides the low-cost production of biochar, it has a number of advantages, including the capacity to reduce secondary environmental contamination, the ability to be recycled, and the ability to produce high-value-added adsorbents^41^. Utilizing biochar as an adsorbent also has the additional benefit of reducing the quantity of carbon dioxide discharged into the environment^42,43^. The fact that the surface of biochars has various important groups on their carbon skeleton, despite the fact that their surface areas and pore volumes are both much lower than those of AC, demonstrates that they are a more efficient source of carbon^44^.

To increase the experimental uses of biochars for the treatment of colorants in aquatic environments, the functional group number on biochars’ surfaces needs to be increased. This can be accomplished through chemical alterations to the surfaces of biochars. It is common knowledge that the adsorption capacity of biochar can be increased by modulations such as metal impregnation, oxidation, nanoscale creation, and carbon surface activation^45^. It is possible to boost the capability of biochar by impregnating it with mineral elements such as amino groups^46^, which increases the removal capacity of the biochar. The oxidation approach of biochar surface increased the functional group number by treatment of biochars with different bases (NaClO, KOH, H_2_SO_4_, H_3_PO_4_, or HNO_3_), acids (H_2_SO_4_, H_3_PO_4_, or HNO_3_), and oxidising reagents (NaClO, H_2_O_2_, NH_3_^.^H_2_O, KMnO_4_, or (NH_4_)_2_ S_2_O_8_)^47–50^. Biochars are loaded with nanometals to support nanoscale metals, improving thermal stability, adsorption sites, specific surface area, and oxidation resistance. It is believed that this will increase the affinity of biochar and make it easier to remove pollutants from water^51^. The interaction of biochars with nitrogen-containing primary, secondary and tertiary amines functional groups as well as the treatment with imidazole and quaternary ammonium led to a biochar surface modification^30–33^. It can be demonstrated that the most frequently utilised reducing agents are Na_2_SO_3_, H_2_, NH_3_^.^H_2_O, FeSO_4_, and aniline^52^. The adsorption of numerous pollutants on these adsorbents, which are made from residual agricultural biomass activated carbon, has been widely researched in the literature. Coconut Shell^53^, Gulmohar^54^, rice straw^55^, orange peel^56^, bamboo^57^, mandarin peel^58,59^, potato^60,61^, wheat straw^62^, sesame shells^63^, olive stone^64^, coffee bean husks^65^, peanut husk^66^, tea waste^67^, watermelon peel^31^ and Macore fruit^68^ are some of these biomasses.

The mandarin is a citrus fruit that grows in temperate climates and is a member of the citrus family in general. Following the data of the FAO organization, the yearly output of mandarin oranges is approximately 21 million tonnes, according to figures published in 2013. Countries with the highest levels of production include China, Brazil, Turkey, Spain, Egypt, Japan, South Korea, and Italy. China is the world’s largest producer of soybeans. Approximately 8–14% of the total weight of mandarins used in fruit juice manufacturers is made up of peels that are dumped into the environment after use. They are mostly utilised in the fertiliser, fuel and animal feed industries, as well as the cosmetics industry^58^. Because of the widespread use of mandarins, a substantial volume of fruit peel is generated as biomass waste^69^. The presence of organic compounds such as cellulose, hemicellulose, and pectin in the composition of mandarin waste materials makes it possible to produce environmentally beneficial biochars from the peel through pyrolysis, which is environmentally friendly. It is possible to obtain materials that have a high adsorption capacity in this manner^70^. However, there has not been a thorough examination into the influence of the Physico-Chemical characteristics of biochar generated from mandarin peel on the elimination of colourants in wastewater has been reported.

There have been no studies made on the adsorption efficiency of biochar produced from mandarin peel (MP) waste materials by first dehydrating with 80% H_2_SO_4_, then oxidising with ozone, and finally aminating with Triethylenetetramine (TETA), as a posible material for the adsorption of AY11 dye from aquatic environments. An inexpensive agricultural waste material, MP, was used to prepare Mandarin-Biochar-TETA (MBT), which was tested for its efficacy in removing Acid Yellow 11 dye from water. As part of the investigation of the removal conditions of AY11 dye from the aquatic environment, variables such as the beginning concentration of AY11 dye, the pH of the aquatic environment, the time of reaction between the MBT and AY11 dye, and the influence of the dose of MBT were explored. The isotherm and kinetic models for AY11 dye adsorption by MBT were studied to identify the adsorption mechanism as well as the *Q*_m_.

## Equipment and materials

### Materials

**Figure 1:**
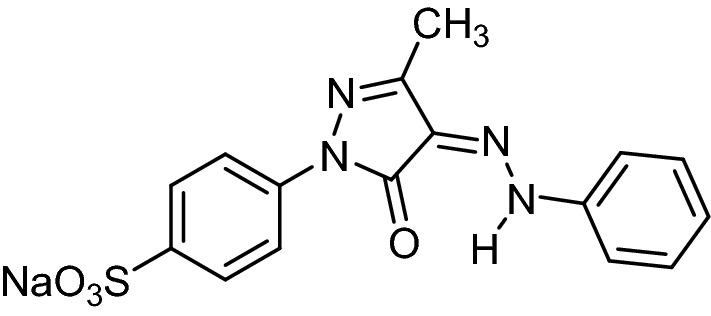
Chemical structure of AY11 dye (MF: C_16_H_13_N_4_NaO_4_S) (MW: 380.35 g/mol) (C.I.18820) (Other names: Acid Yellow G, Acid Brilliant Yellow 2R, Acid Brilliant Yellow G) (CAS number: 6359-82-6).

Mandarin (*Citrus reticulata*) peels raw material used for the formation of MBT was obtained from the local market in Alexandria, Egypt. Concentrated 99% Sulfuric acid (H_2_SO_4_), TETA and Acid Yellow 11 (AY11) dye (MF: C_16_H_13_N_4_NaO_4_S) (Fig. 1) were importent from Sigma Aldrich, USA. 1000 mg L^–1^ stock solution of AY11 dye was prepared using AY11 dye (one gram) in distilled water (one liter). Pg equipment model T80 UV/Visible high-performance double beam spectrophotometer matched to a 1 cm optical path glass cells, an electric shaker (model JSOS-500) and pH equipment (model JENCO-6173) were applied.

### Formation of the MBT

Mandarin (*Citrus reticulata*) peel was washed with distilled water and dried at 85 °C for 72 h in an oven. Approximately 200 g of dried mandarin peels (in 1.0 L of 80% H_2_SO_4_ in a refluxed system) were boiled for 3 h at 200 °C, then treated with three letres of distilled water, filtered and repeatedly washed with water to have a neutral filtrate, and washed once with ethanol followed by dried for 24 h at 120 °C in an oven and then weighed to yield 85 g. The dehydration material was oxidised for 60 min in distilled water after being treated with ozone for 30 min^30,48^. It was necessary to weigh 65 g of oxidised mandarin biochar after it had been filtered, washed with distilled water, and dried at 120 °C for 24 h in an oven. A solution of TETA (80 mL) was added to the 25 g of oxidised mandarin biochar, and the mixture was heated in a soxhlet system for 2 h. Afterwards, the modified biochar product was filtered off, washed with distilled water, and followed by ethanol washing twice before being dried at 120 °C for 24 h to yield 28 g of the surface-modified biochar, which was labelled as MBT.

### Experiment of batch adsorption

AY11 dye solution (1000 mg L^–1^) was prepared as a stock solution for the experimental work and was subsequently diluted to the necessary calibration standard curve concentrations and to the experimental concentration tests. For the purpose of determining the adsorption capacities, thermodynamic, and kinetic properties of the MBT, batch experimental tests were used. At room temperature (RT), a series of Erlenmeyer flasks (250 mL) having 100 mL of various concentrations of AY11 dye solution and varying masses of MBT was agitated at 200 rpm for a predetermined period until the dye solution color was completely constant (25 experiment work was conducted). A 0.1 M HCl solution or a 0.1 M NaOH solution were used to alter the pH of the sample to the appropriate levels, respectively. A 1 mL sample solution was collected at interval times from the Erlenmeyer flask and centrifugated to separate the adsorbent from the solution, and the concentration of the AY11 dye was measured by spectrophotometer equipment (*λ*_max_ = 407 nm). Due to the need for simplicity, the adsorption operations were repeated three times, and only the average values were used in the thermal and kinetic analyses. Equation (1) was used to calculate the equilibrium adsorption capacities (*q*_e_) of the samples:

1 $${q}_{t}=\frac{\left({C}_{0}-{C}_{t}\right)}{W}\times V.$$

*q*_t_ (mg AY11 dye/g MBT) (adsorption capacity) is the ability of the adsorbent to remove AY11 dye from a solution at a specific time is expressed as mg AY11 dye/g MBT. In this equation, *C*_0_ (mg L^–1^) represents the beginning concentration of AY11 dye, and *C*_t_ (mg L^–1^) represents the residual concentration of AY11 dye after the adsorption process has been completed for a specified period of time (in minutes). If you want to know how much AY11 dye has been eliminated from an aqueous solution, you can use the following Eq. (2).

2 $$Removal\left(\%\right)=\frac{\left({C}_{0}-{C}_{t}\right)}{{C}_{0}}\times 100.$$

The impact of pH on the removal of AY11 dye was studied by agitated of 0.025 g of MBT and 100 mL of AY11 dye solution at pH values ranging from 1.5 to 12. The pH levels were fixed to the desired pH using 0.1 M HCl and 0.2 M NaOH solutions, and the results revealed that pH had an effect on AY11 dye adsorption. It needed 180 min of agitation at 200 rpm and 25 °C before samples were taken for AY11 dye measurement, which took place after 3 h.

To conduct the isotherm research, AY11 dye (100 mL) was agitated at 200 rpm for 3 h at RT (24 ± 2 °C) with chainging the beginning concentrations of AY11 dye from 100 to 400 mg L^–1^ and varying masses of the MBT from 75 to 175 mg until the dye solutions were completely discoloured, at which point they were discarded. The impact of the MBT dose and reaction time on AY11 dye removal was examined by agitated of 100 mL of AY11 dye of different beginning concentrations and MBT with varying dosages (75, 100, 125, 150, and 175 mg) at different reaction times at RT. All experimental work was repeated three times and only the mean value was recorded and used in this work.

### MBT characterization

Isotherm of the adsorption–desorption curve of the MBT material was estimated using a mathematical model at the boiling point of N_2_ gas. It was determined that the activated biomass-based biosorbent had a BET surface area of (*S*_BET_) when nitrogen was adsorbed at 77 K using the equipment of BELSORP—Mini II, BEL Japan, Inc. When calculating the surface area (*S*_BET_) for the isotherm, the BET plot was used to compute the energy constant (*C*), total pore volume (*p*/*p*_0_) (cm^3^/g), the monolayer volume (*V*_m_) (cm^3^ (STP) g^–1^), the mean pore diameter (nm) for the isotherm curve. The average pore radius was measured with the help of Eq. (3).

3 $$r\left({\text{nm}}\right)=\frac{{2V}_{T}\,({\text{mL}}\, {\text{g}}^{-1})}{{a}_{s,BET} \,({\text{m}}^{2}\,{\text{g}}^{-1})}\times 1000,$$

where *r* (nm) is the average pore radius, *V*_T_ (mL g^–1^) is the total pore volume and *a*_S,BET_ (m^2^ g^–1^) is the calculated specific serface area from BET model analysis. The BJH method developed by Barrett–Joyner–Halenda^71^ was used to determine the *S*_mi_ (micropore surface area), *S*_mes_ (mesopore surface area), *V*_mi_ (micropore volume), and *V*_mes_ (mesopore volume) of the produced MBT material. In the BJH approach, the pore size distribution is estimated from the desorption isotherm. The SEM equipment QUANTA 250 was used to analyse the MBT sample surface morphology and was used in combination with an EDX (Energy Dispersive X-ray Spectrometer) for elemental analysis of MBT. Platinum ATR unit model V-100 connected to Bruker VERTEX70 to make the FTIR (Fourier Transform Infrared) spectroscopy to investigate the surface functional groups of the prepared MBT adsorbent. At temperatures ranging from 50 to 1000 °C with a ramping temperature rate of 5 °C min^–1^, thermal analyses were performed using the SDT650 equipment according to the manufacturer’s specifications.

## Results and discussions

### The MBT characteristics

**Figure 2:**
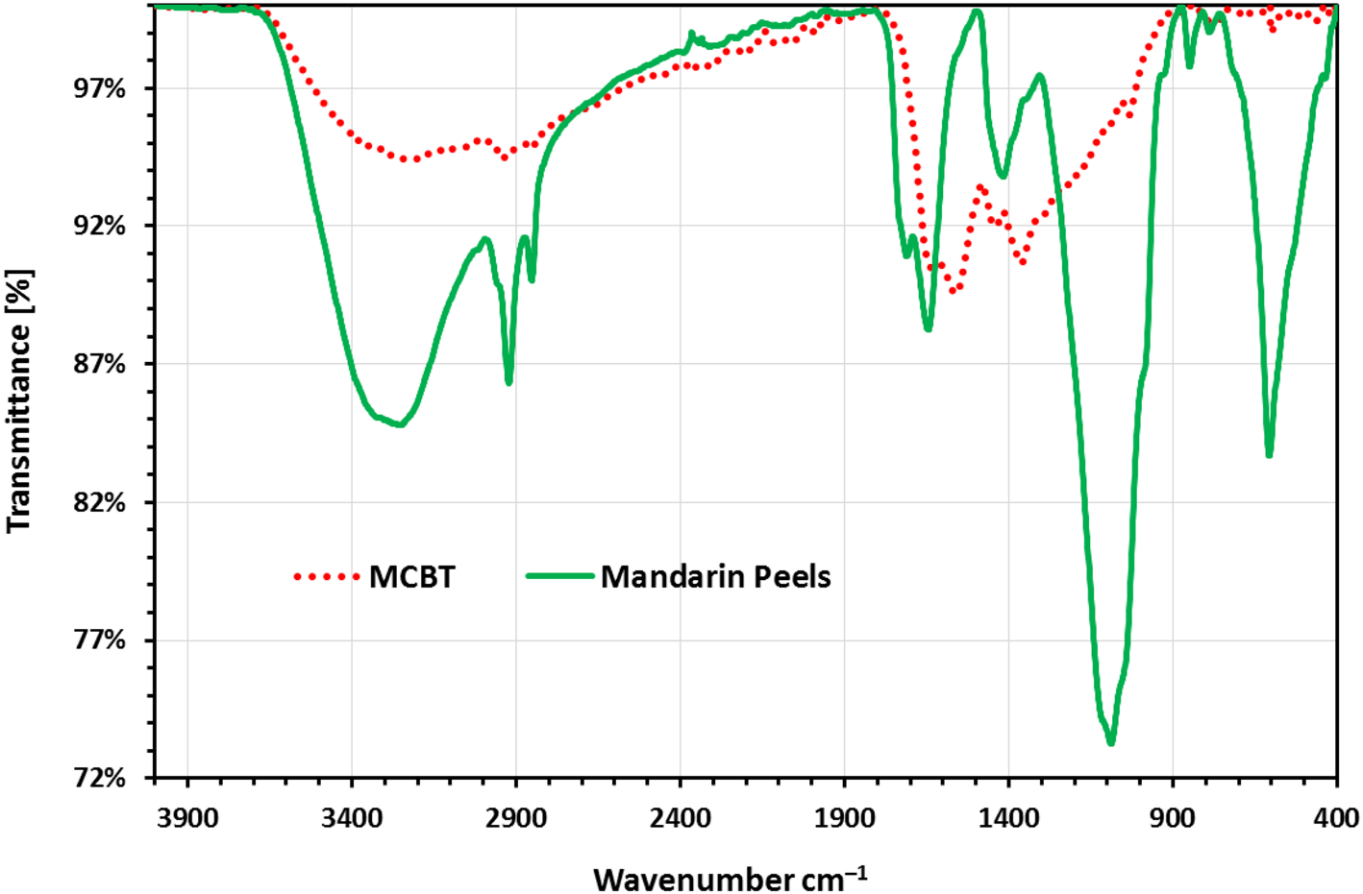
FTIR analysis of MP (green line) and MBT (red doted).

The activated biomass-based biosorbent sample was analysed using FTIR (Fourier Transform Infrared) Spectroscopy analysis method to find alterations in the surface functional groups of the prepared MBT material. The FTIR spectra of raw MP and MBT adsorbent are presented in Fig. 2. Furthermore, while the strong adsorption peak at 3253.1 cm^–1^ represents the O–H groups found in MP, the broad adsorption band at 3239.5 cm^–1^ suggests that the prepared MBT contains –OH and –NH groups of the glucose moiety and the amino group, respectively (Fig. 2). That a new band had formed meant that the amino group had been incorporated into the structure of the activated biomass-based biosorbent. In mandarin peels, a stretching vibration of –CH_2_ was noticed, and the MBT was found at 2923.6–2855.2 and 296.1–2858.2 cm^–1^, respectively. The band at about 1711 cm^–1^ is due to the C=O vibration of the carboxyl group that appeared in MP but did not appear in the produced aminated biosorbent (Fig. 2). The peak at 1645 cm^–1^ in MP and 1633.1 cm^–1^ in MBT suggests that both contain amide groups. TETA alteration may have enhanced MBT’s N–H functional group by increasing the frequency of the N–H vibration in the fatty amine or the aromatic secondary amine, which occurs at 1560 cm^–1^ in MBT. A substantial adsorption peak at 1420 cm^–1^ was seen in MP, but the high adsorption bands at 1441 and 1364 cm^–1^ in MBT were attributed to the –N=C=O group vibration. This new peak on MBT, which was created by functional groups containing nitrogen, suggests that amino groups were successfully formed onto the surface of MBT as a result of TETA treatment. In MBT, the C–O–H group is proved by the peak at 1036 cm^–1^, but in mandarin peels, the C–O–H group is proved by the peak at 1093 cm^–1^. Furthermore, the peak strength in the range of 1029–1092 cm^–1^ showed a significant difference between MP and MBT, implying that TETA alterations could affect the C–O–H functional group of MBT (Fig. 2). Furthermore, in the newly designed adsorbent MBT, the OH group vibration that occurred at 606 cm^–1^ in MP was completely eliminated.

**Figure 3:**
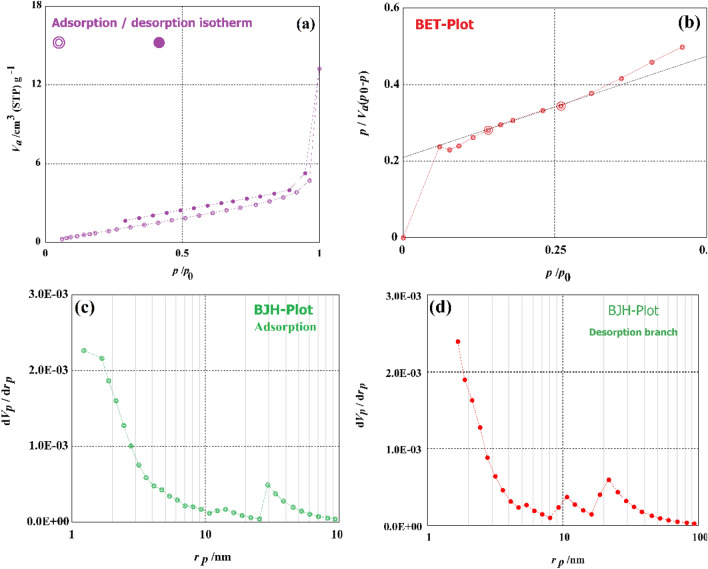
(**a**) Adsorption–desorption graph of MCT, (**b**) BET analysis graph of MCT, and (**c**) BJH adsorption analysis graph of MCT; (**d**) BJH desorption analysis graph of MCT.

The textural characteristics of the MBT were measured using the BET and BJH analyses (Fig. 3). This method is applied to determine the specific surface area (*S*_BET_), mean pore diameter, total pore volume, monolayer volume, mesopore volume, mesopore area, and mesopore distribution. MBT has a BET-specific surface area of 5.48 m^2^ g^–1^ and a monolayer volume of 1.3316 cm^3^(STP) g^–1^ in terms of surface area. The total pore volume of MBT was 0.016 cm^3^ g^–1^, with a mean pore diameter of 11.612 nm and a mean pore volume of 0.016 cm^3^ g^–1^ (mesopores). MBT has a mesopore volume of 0.01722 cm^3^ g^–1^ and a mesosurface area of 6.0664 m^2^ g^–1^. With a length of 1.22 nm, MBT showed a mesopore distribution peak. The amine functional groups in the MBT generated are thought to be responsible for shutting the pores^30–33^.

**Figure 4:**
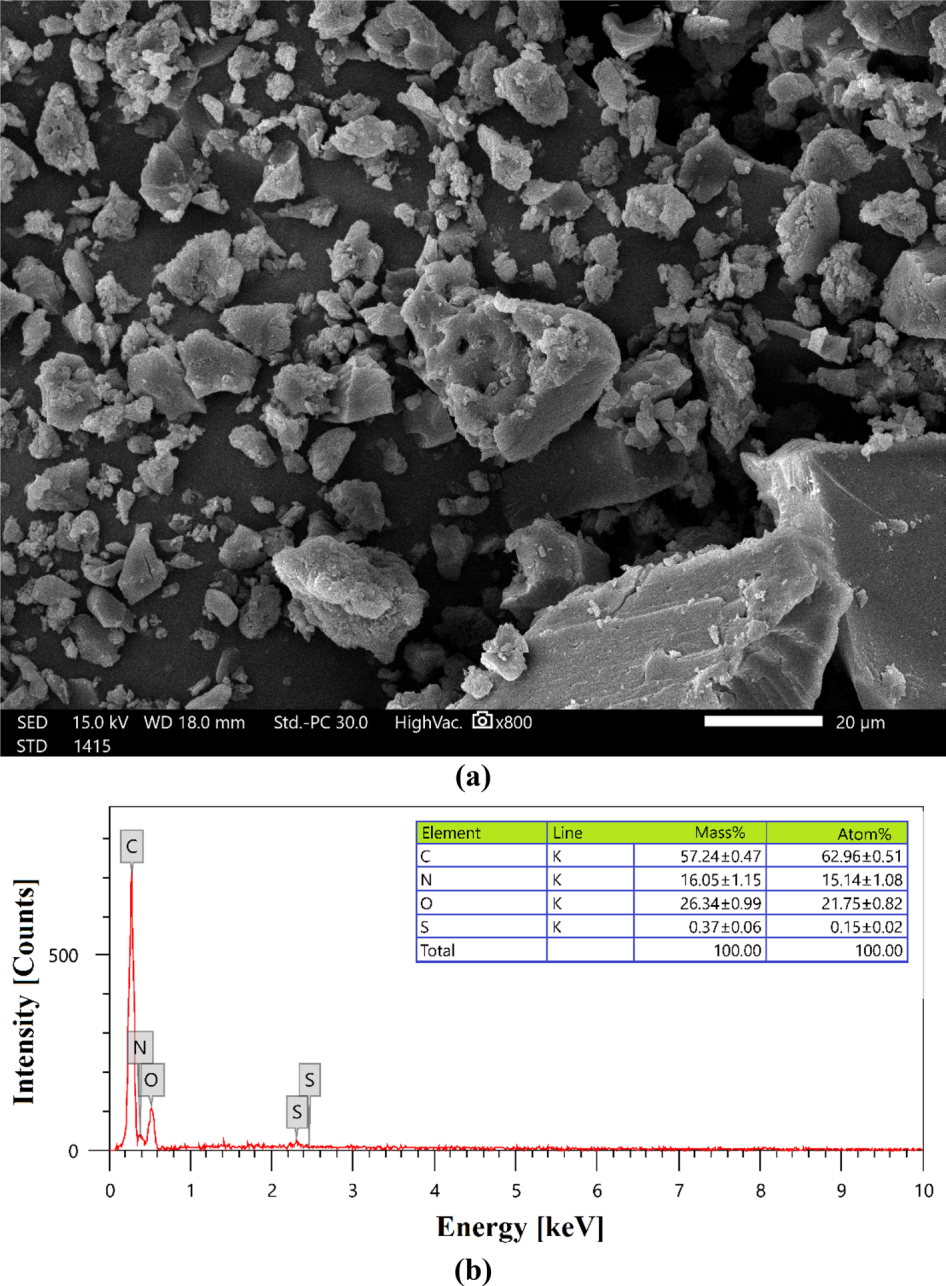
(**a**) SEM image of the MBT magnification at × 800 under high vacuum; (**b**) EDX analysis of MBT prepared from mandarin peels.

SEM (Scanning Electron Microscope) was used to analyse the MBT. The amine had closed the majority of the pores and caves, resulting in the development of active sites^30–33^. Figure 4a displays the MBT surface morphology, which shows that the newly introduced amine group had closed the majority of the pores and caves, resulting in the production of active sites. It was discovered through image characterization that the surface had an irregular shape, uneven edge aggregation, and a nonporous surface, all of which might have resulted from surface amination with TETA^72^. According to the BET analysis, the surface area produced from this nonporous surface structure is smaller than expected.

The EDX analysis of the MBT was performed in order to determine its chemical makeup. According to Fig. 4b, the MBT chemical composition was obtained by EDX analysis, which revealed that the element nitrogen accounted for 16.05% of the total sample weight in the compound. Carbon atoms accounted for the majority of the elements in the MBT (57.24 ± 0.47%), oxygen atoms (26.34 ± 0.99%) and nitrogen atoms (16.05 ± 1.15%). As a result of the dehydration process with 80% H_2_SO_4_, a tiny number of sulphur atoms (0.37 ± 0.06%) were detected in the MBT.

**Figure 5:**
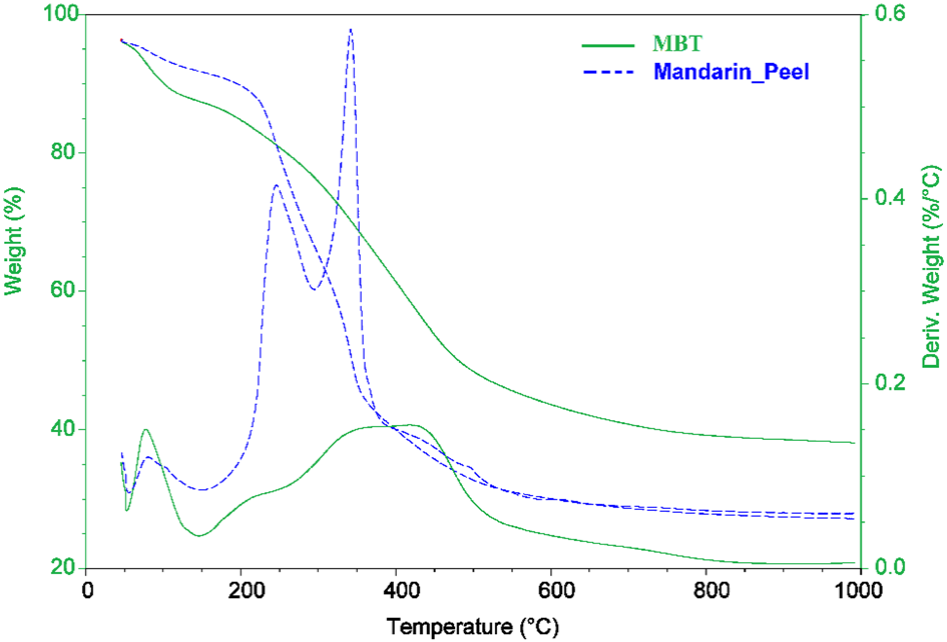
The thermogravimetric curves of the MP and MBT using TGA and DTA.

The thermogravimetric curves of the MP and MBT as a function of temperature are presented in Fig. 5. Unlike the raw material MP, which decomposes in 4 steps, the MBT decomposes in 2 processes, as presented in Fig. 5. Due to the loss of water and moisture bounded to the surface of the MP and MBT in the first step, which takes place between 50 and 150 °C, the sample loses between 4.5 and 8.85%, respectively, of its weight. Mandarin peels lost 25.27% of their weight at 150–300 °C, and MBT lost 49.33% of its weight at 150–1000 °C in the second stage of weight loss. The MP loses about 24.94% of its weight in the third stage of the weight-loss, which occurs between 300 and 385 °C, and 14.23% in the final (the fourth) weight-loss stage, which occurs at a temperature between 385 and 1000 °C^30–33^.

Despite the fact that it can be used only for identification, differential thermal analysis (DTA) is most typically utilised for investigation of the phase diagram, measurements of heat change, and breakdown in a variety of atmospheres (Fig. 5). The mandarin-peels sample’s DTA curve shows three peaks at flow Tf temperatures (80.20, 243.72, and 342.51 °C), with the greatest peak at 80.20 °C. The pyrolysis of the mandarin peels, on the other hand, produces three distinct degradation peaks that may be distinguished. The DTA analysis of the MBT indicated only two well-resolved decomposition bands at temperatures flow Tf (78.53 and 418.75 °C) as well as start points at 57.9 and 213.15 °C. This demonstrated that the MBT sample stability might be improved by modification rather than mandarin peels^30–33^.

The most significant reactions of O_3_ with organic matter are likely based on the cleavage of the carbon double bond, which acts as a nucleophile having excess electrons^72^. For example, the injected O_3_ air stream may, to some extent, lead to the formation of carbonyl and carboxyl groups on biochar surfaces by reacting with certain C=C double bonds of biochar materials:

4 $${\text{Biochar}}{-}{\text{CH }} = {\text{ CH}}{-}{\text{Biochar }} + {\text{ O}}_{{3}} \to {\text{ Biochar}}{-}{\text{COH }} + {\text{ Biochar}}{-}{\text{COOH}}.$$

The ozonized biochar product will (A) become more hydrophilic since both carbonyl and carboxyl groups can attract water molecules; and (B) have higher Biochar cation exchange capacity (CEC) value since the carboxyl groups readily deprotonate in water and result in more negative charge on the biochar surfaces:

5 $${\text{Biochar}}{-}{\text{COOH }} \to {\text{ Biochar}}{-}{\text{COO}}^{ - } + {\text{ H}}^{ + } .$$

### Acid Yellow 11 dye adsorption on MBT

#### Impact of pH

**Figure 6:**
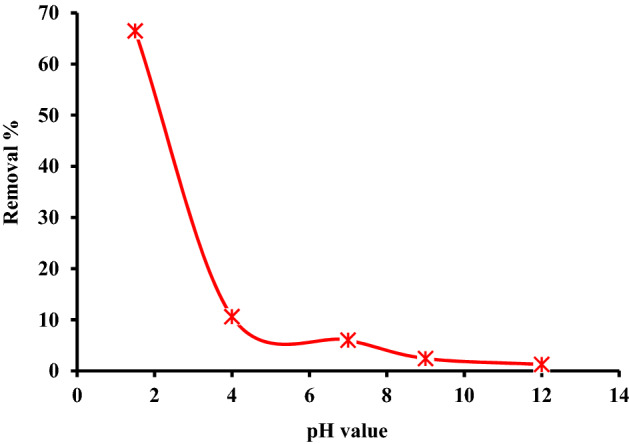
Removal of AY11 dye by MBT as a function of pH as a function of removal% (AY11 dye = 75 mg L^–1^, MBT = 0.025 g L^–1^, temperature = 24 ± 2 °C).

The pH of wastewater from the textile sector varies greatly. The solution pH has a high impact on the process of adsorption because it influences the hydroxyl, carboxyl, and amino groups on the surface of MBT biochar. At RT (24 ± 2 °C), using 0.025 g L^–1^ of MBT as an adsorbent and a beginning concentration of AY11 dye (75 mg L^–1^), the equilibrium adsorbed quantity (*q*_e_) of the Acid Yellow 11 (AY11) dye was measured and this dye was removed for three hours, the adsorption of AY11 dye was examined at pH ranging from 1.5 to 12, and the curve of the pH variations is shown in Fig. 6. The maximum removal (66.5%) of AY11 dye has occurred at pH 1.5 for AY11 dye removal using MBT, as shown in Fig. 6. The pH was repeatedly reduced from 1.5 to 12 in the AY11 dye removal trial, causing the adsorption rate to drop from 66.5 to 1.3%. The drop in adsorption elimination percentage was most pronounced between pH values of 1.5 and 4, but only modestly between pH values of 4 and 12. In their Direct Yellow 12 (DY12) dye removal investigation, Khaled et al.^73^ discovered that by increasing the solution pH from 1.5 to 11.1, the adsorption effectiveness reduced from 98.1 to 11.1%. Furthermore, in the Methyl Orange (MO) dye adsorption, increasing the pH from 2 to 11 lowered the adsorption effectiveness from 98 to 56%, according to Aboua et al.^68^. When Sunset Yellow dye was removed, Song et al.^35^ found that the pH value increasing from 2 to 4 significantly lowered the capacity of the adsorption. The best pH for removing AY11 dye with MBT was found to be 1.5.

Excess HO^–^ ions in a high pH value solution (alkaline) lower removal effectiveness by competing for adsorption sites with the AY11 dye ions, an anionic dye. Furthermore, the MBT adsorbent prefers to absorb OH ions that have a higher concentration and mobility than dye anions. The electrostatic attractive forces that arise as the positively charged areas increases in acidic medium aid in the adsorption of anions. The exceptionally high adsorption effectiveness at the strongly acidic pH 1.5 zone can be discussed as follows: in reality, surface areas on MBT of negatively charged do not enable anionic AY11 dye adsorption because of electrostatic repulsion. This is because biochar is hydrophobic. When the adsorbent MBT is submerged in water, hydrogen atoms bond to the carbon surface which positively charges the MBT. The development of attractive contacts between the negatively-charged AY11 dye and the positively-charged MBT enables dye removal.

#### Impact of reaction time

**Figure 7:**
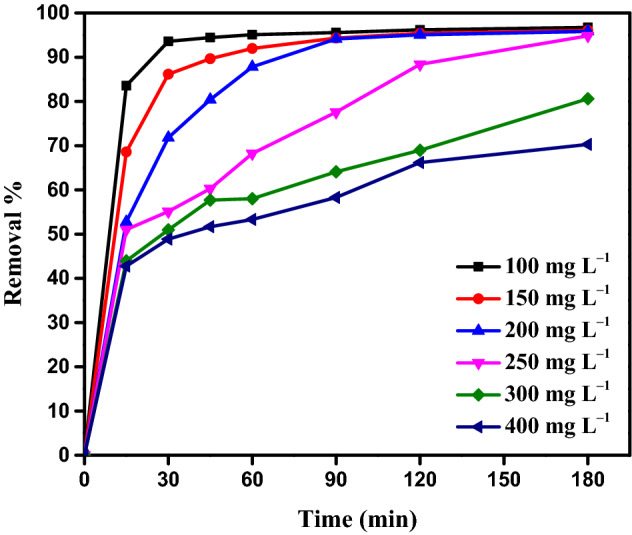
The removal of AY11 dye for 3 h using MBT as an adsorbent (*C*_0_ of AY11 dye: 100–400 mg L^–1^, MBT dose = 0.75 g L^−1^, RT = 24 ± 2 °C).

Time is crucial for MBT and AY11 dye to form the required contact. As a result, the influence of contact duration on MBT at pH 1.5 and AY11 dye concentrations ranging between 100 and 400 mg L^–1^ was examined. The adsorption process happens quite quickly in the first 15 min, and then progressively increases beyond that minute, as seen in Fig. 7. Figure 7 reveals that the first 15–30 min account for 43–94% of the AY11 dye adsorption. The clearance of AY11 dye increased linearly with increasing contact time, reaching 96.74, 96.06, 95.84, 94.86, 80.61, and 70.29% after 3 h, depending on the beginning concentration of AY11 dye (100 to 400 mg L^–1^).

Because of the low concentration of dye in the empty active sites, the majority of these ions will adhere to the MBT adsorbent, allowing for a considerable distance to be covered while eliminating AY11 dye at a low amount of the MBT adsorbent dose. When AY11 dye is removed by the MBT adsorbent at a high beginning dye concentration, the removal percentage remains low because the active sites are unable to adsorb new dyes after being loaded with a particular number of AY11 dyes. El-Nemr et al.^32^ identified a similar pattern in their study of the AY11 dye adsorption.

#### Impact of AY11 dye starting concentration

**Figure 8:**
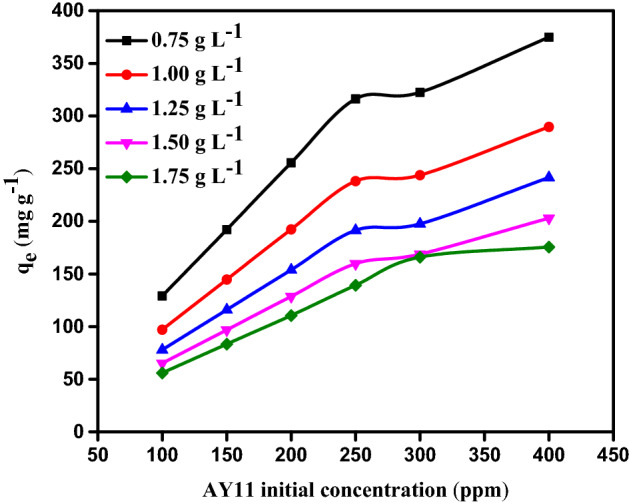
At Temp. (25 ± 2 °C), the effect of AY11 dye starting concentration (100–400 mg L^–1^) utilising MBT dose (0.75–1.75 g L^–1^) on *q*_e_ (mg g^–1^).

To investigate the impact of the starting concentration of AY11 dye on the equilibrium adsorption capacity, the MBT concentration (0.75, 1.00, 1.25, 1.50, and 1.75 g L^–1^) and the AY11 dye starting concentration (100, 150, 200, 250, 300, and 400 mg L^–1^) were investigated at RT (24 ± 2 °C) at pH 1.5 and also to assess the impact of MBT dosage on the equilibrium adsorption capacity (*q*_e_). According to Fig. 8, the amount of AY11 dye adsorbed rises with decreasing MBT dosages for the same starting concentration of AY11 dye at equilibrium (*q*_e_). As shown in Fig. 8, equilibrium adsorption capacities (*q*_e_) for the removal of AY11 dye were measured using MBT at various doses (0.75–1.75 g L^–1^). These values ranged from 128.9 to 374.8 mg g^–1^ for starting AY11 dye concentrations (100, 150, 200, 250, 300, and 400 mg L^–1^), 97.0 to 289.6, 77.9 to 241.5, 65.1 to 202.9, and 55.9 to 175.5 mg g^–1^ for starting concentrations of AY11 dye (100, 150, 200, 250, 300, and 400 mg L^–1^). At equilibrium, the adsorption capacity (*q*_e_) of AY11 dye by MBT is greater in solutions with a higher starting AY11 dye concentration, as illustrated in Fig. 8. It was noticed that as the adsorbent dose was raised, the *q*_e_ value decreased. As a result, it appears as though the adsorption of the AY11 dye from its water solution is controlled by its starting concentration. Khaled et al.^73^ saw a similar trend in their work on the eradication of DY12 dye. In the removal approach of AY11 dye by MBT, AY11 dye molecules first overcome the boundary layer impact, then diffuse out of the film of the boundary layer on the surface of MBT, and at the end are bound to the porous structure of the MBT.

#### The impact of MBT dose on the AY11 dye adsorption

**Figure 9:**
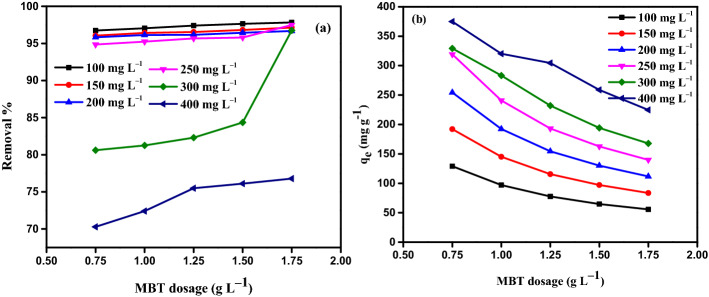
The impact of different MBT doses (0.75–1.75 g L^–1^) of AY11 dye different starting concentration (100–400 mg L^–1^) at RT = 24 ± 2 °C (**a**) on percentage of removal; (**b**) on *q*_*e*_ (mg g^−1^).

To assess the influence of MBT dose on AY11 dye adsorption, the starting concentration of AY11 dye (100–400 mg L^–1^), MBT doses (0.75–1.75 g L^−1^), solution temperature (24 ± 2 °C), contact period (180 min), and pH of the solution were all fixed at 1.5. (Fig. 9). The percentage of AY11 dye adsobent (percent) increases slightly with increasing in the MBT dose (ranging from 94.86 to 96.74%) (Fig. 9a) for starting concentrations of 100–250 mg L^–1^ AY11 dye (from 80.61 to 96.76% for the 300 mg L^–1^ starting concentration of AY11 dye and from 70.29 to 76.78% for the 400 mg L^–1^ starting concentration of AY11 dye). Due to the fast filling of the active sites on the surface of MBT in the presence of high dye molecules concentration, the removal% is low in the scenario where the MBT dose is 0.75 g L^–1^ and the starting AY11 dye concentration is 300–400 mg L^–1^. As a result, between 70 and 80% of the colour was eliminated. With increasing MBT dose, the equilibrium adsorbed amount of AY11 dye (*q*_e_) decreases (Fig. 9b).

By increasing the MBT amount from 0.75 to 1.75 g L^–1^, the q_e_ of AY11 dye rises from 128.9 to 55.6, 192.1 to 83.5, 254.3 to 111.6, 319.2 to 139.8, 329.2 to 167.7, and 374.8 to 224.6 mg g^–1^ for starting AY11 dye concentrations of 100, 150, 200, 250, 300, and 400 mg L^–1^, respectively. Using an MBT dose of 1.75 g L^–1^, the highest percent removal of the AY11 dye and the equilibrium adsorption amount (*q*_e_) were measured.

### Adsorption isotherms

Described as a relationship between the *q*_e_ (mg g^–1^) at equilibrium time and the dye concentration (*C*_e_ in mg L^–1^) at the equilibrium time. The adsorption isotherm is used to show the state of adsorbate molecules dispersed across the solid and liquid phases^74,75^. To determine the optimal amount of MBT and the molecular proportion of adsorbate that should be distributed in the equilibrium (*q*_e_) between the solid and liquid phases, isotherm data is used. To investigate the interaction between MBT and AY11 dye, various isotherm models including the LIM (Eq. (6)), FIM (Eq. (7)), TIM (Eq. (8)), and DRIM (Eq. (9)) were employed^74^.

6 $$\frac{{C}_{e}}{{q}_{e}}=\frac{1}{{Q}_{m}\times {K}_{L}}+\frac{{C}_{e}}{{Q}_{m}},$$

7 $${\text{log}}\left({q}_{e}\right)={\text{log}}\left({K}_{F}\right)+\frac{1}{n}\times {\text{log}}\left({C}_{e}\right),$$

8 $${q}_{e}=\frac{RT}{b}{\text{ln}}\left({K}_{T}\right)+\frac{RT}{b}{\text{ln}}\left({C}_{e}\right),$$

9 $${\text{ln}}\left({q}_{e}\right)={\text{ln}}\left({q}_{m}\right)-K{\varepsilon }^{2}.$$

**Figure 10:**
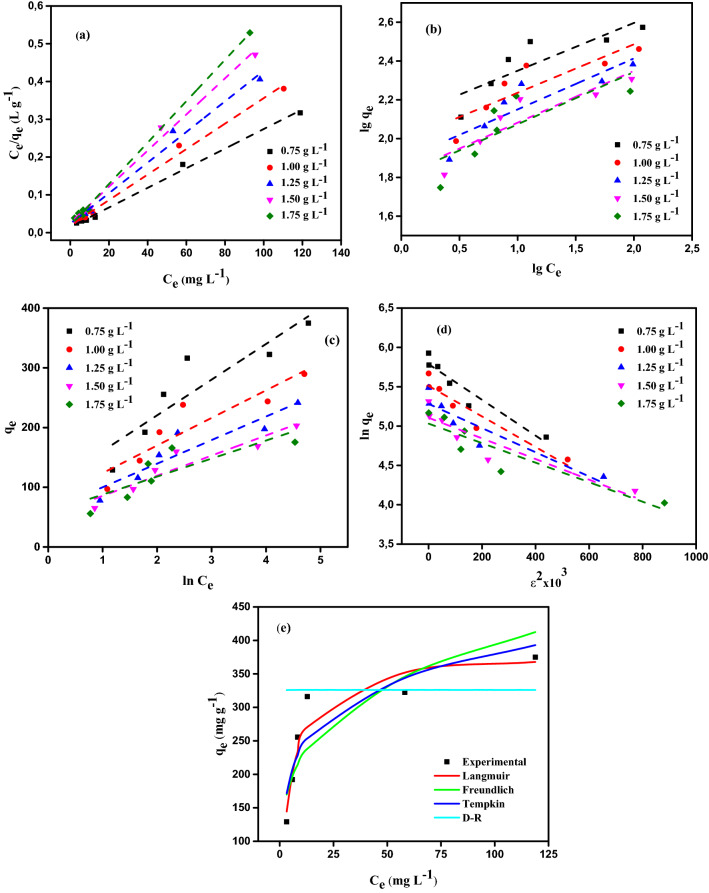
(**a**) Linearized Langmuir, (**b**) Freundlich, (**c**) Temkin, (**d**) Dubinin-Radushkevich isotherms profiles for AY11 dye at (25 ± 2) °C at starting concentrations (100–400 mg L^–1^) on MBT doses (0.75–1.75 g L^–1^). (**e**) When AY11 dye was used at a starting concentration of 100–400 mg L^–1^ on an MBT dose of 0.75 g L^–1^, the isotherm profiles were compared between the measured and predicted isotherms profiles at RT (24 ± 2 °C).

The calculated data as a consequence of the removal of AY11 dye by MBT are reported in Table 1, where the Langmuir isotherm model constants are adsorption site affinity (*K*_L_) and saturated monolayer adsorption capacity (*Q*_m_). MBT dose of 0.75 g L^–1^ showed a good correlation coefficient (*R*^2^ 0.990) in the linear type of the LIM after the AY11 dye was removed, and the maximal monolayer capacity (*Q*_m_) was measured to be 384.62 mg g^–1^. The 1/*Q*_m_*K*_L_ and 1/*Q*_m_ values of the LIM are indicated by the intercept and slope of the *C*_e_/*q*_e_ vs *C*_e_ plot in Fig. 10a, respectively. The adsorption of AY11 dye on MBT can be supported by a high correlation coefficient (*R*^2^ 0.990) and constants of the equilibrium adsorption (*K*_L_) ranging between 0.18 and 0.29 L mg^–1^. Following the Langmuir isotherm model, the AY11 dye could be used for adsorption on MBT. As a result, it was determined that the AY11 dye was adsorbed as a monolayer on the MBT.

**Table 1:** Data from an isotherm investigation of AY11 dye adsorption using MBT [AY11 dye starting concentration (100–400 mg L^–1^), MBT dose (0.75–1.75 g L^–1^), and RT (24 ± 2 °C)].

Isotherm model	Parameters	MBT dose (g L^–1^)				
		0.75	1.00	1.25	1.50	1.75
LIM	*Q*_*m*_ (mg g^-1^)	384.62	294.12	243.90	208.33	181.82
	*K*_*L*_ × 10^3^	0.18	0.18	0.18	0.19	0.29
	*R*^2^	0.995	0.993	0.990	0.993	0.998
FIM	1/*n*	0.247	0.250	0.263	0.267	0.269
	*Qm* (mg g^–1^)	368.09	306.62	274.72	249.09	124.29
	*K*_*F*_ (mg^1–1/n^ L^1/n^ g^–1^)	126.77	96.78	77.16	65.30	30.82
	R^2^	0.745	0.760	0.793	0.815	0.613
TIM	*A*_*T*_	5.422	5.379	4.642	4.657	6.723
	*B*_*T*_	59.747	46.163	39.468	33.723	30.185
	*R*^2^	0.825	0.840	0.863	0.890	0.675
DRIM	*Q*_*m*_ (mol kg^–1^)	326.10	247.27	196.70	164.93	153.10
	*K* × 10^6^ (mol kJ^–1^)^2^	2.3	1.9	1.5	1.3	1.2
	*E* (kJ mol^–1^)	466.25	512.99	577.35	620.17	645.50
	*R*^2^	0.916	0.910	0.862	0.860	0.835

The FIM is another model that was applied for the AY11 dye adsorption by MBT adsorbents. To see how successful MBT was at removing AY11 dye, the Freundlich isotherm model was used. Table 1 reports the parameters calculated from the linear fitting of the FIM, which represents the process of adsorption as a heterogeneous phenomenon. The log*K*_F_ and 1/*n*_F_ values of the Freundlich isotherm model are indicated by the intersection and slope of the plot of log(*q*_e_) against log(*C*_e_) as displayed in Fig. 10b. The significance of *K*_F_ (L g^–1^), which expresses the AY11 dye amount removed by the unit of equilibrium concentration adsorbent and is one of FIM constants as a partition or coefficient of adsorption, is that it expresses the AY11 dye amount removed by the unit of the adsorbent equilibrium concentration. The higher the *K*_F_ value, the greater the capacity of the adsorption. Furthermore, a ratio of 1/*n* smaller than 1 indicates that the adsorbent may easily absorb the adsorbate.

As a result, when 1/*n* is less than 1, MBT uses a physical procedure to remove the AY11 dye. Because all of the values of 1/*n* in Table 1 are less than one, it can be assumed that the AY11 dye adsorption using the MBT is good. Also, the *n*_F_ value showed the degree of non-linearity between the solution concentration and the adsorption process, and when 1/*n* value is more than 1, the AY11 dye is physically adsorbing onto the MBT.

The values of the Freundlich isotherm correlation coefficient are effectively defined by the variation of log (*q*_e_) as a function of log (*C*_e_), as shown in Fig. 10. The high *Q*_m_ value of AY11 dye on the MBT, which is 368.09 mg g^–1^, and the fact that it belongs to the MBT with a 0.75 g L^–1^ concentration is responsible for the improved adsorbability of the AY11 dye on the MBT, as reported in Table 1. When comparing MBT and Langmuir correlation coefficients, the Freundlich correlation coefficient (*R*^2^ = 0.613) was lower than the latter.

Another isotherm model, the Temkin model, has been studied and proved to be accurate. It tackles the impacts of indirect adsorbent/adsorbate contact on the removal process. In the TIM, heat exchange occurs between the AY11 dye and the MBT surface during the adsorption of the dye on the MBT surface. It is expected that the adsorption heat of all dye molecules in the layer will linearly decrease with time in order to anticipate how long it will take for all molecules in the layer to adsorb. Based on the linear relationship between qe and ln*C*_e_ shown in Fig. 10c, the TIM parameters (*A*_T_ and *B*_T_) of AY11 dye adsorption by MBT are calculated from the data obtained from this experiment. The equilibrium binding constant *A*_T_ (g L^–1^) is calculated from the slope of the curve, and the heat coefficient of adsorption (*B*_T_) is measured from the intercept of the curve, which is reported as a percentage of the equilibrium binding constant. The derived Temkin model constants are listed in Table 1. Because of the low Temkin isotherm correlation coefficient (*R*^2^ = 0.675) obtained when removing the AY11 dye using 1.75 g L^–1^ MBT dosage, the TIM is not recommended for investigating temperature differences in the process of adsorption, and it is proved that the TIM is not appropriate for researching temperature differences. With the extremely low heat of sorption and the extremely weak ionic contact between the dye and the MBT, the AY11 dye was eliminated by physisorption and the adsorbate was removed from the solution. The heat of adsorption (*B*_T_) is linked to the adsorbent-adsorbate interaction, and the coating of the AY11 dye on the MBT is altered by this heat, which is measured in degree Celsius. Table 1 shows that this value decreased continually as the MBT dose increased from 0.75 to 1.75 g L^–1^.

In the Dubinin-Radushkevich (DRIM) isotherm model, adsorption data at equilibrium are used to study whether the adsorption of the AY11 dye onto the MBT is chemical or physical in nature. It is named after Dubinin and Radushkevich, who developed the model. When the Polanyi potential theory is taken into consideration, it is expected that the removal process will continue until all of the pores have been entirely filled in. Following the removal of the AY11 dye by the MBT at various doses, the *R*^2^ and DRIM constants were computed and reported in Table 1. For the purpose of determining the kind of adsorption, the binding energy (*E*) value is used. When looking at the estimated *E* values in Table 1, it can be shown that all MBT dosages have *E* value larger than 16 kJ mol^–1^, indicating that the adsorption of AY11 dye on MBT is chemical^76,77^. AY11 dye elimination from MBT at different doses in the DRIM was observed to have correlation coefficients (*R*^2^) ranging between 0.835 and 0.916. These values appear to be better with respect to most adsorbent doses when compared to Freundlich and Temkin isotherms models, which are more consistent with the experimental data (Fig. 10d; Table 1). Figure 10e shows the difference between the four distinct isotherm models that were utilised in conjunction with experimental data.

### Examinations of the error function for the best-fit isotherm model

It was necessary to compare the correlation coefficients (*R*^2^) of the LIM, FIM, TIM, and DRIM with those of the experimental equilibrium data in order to identify which model was the most appropriate for AY11 dye adsorption to MBT. When determining the appropriate isotherm model for experimental data, it’s also useful to examine different error function values. To investigate the error function distribution between the experimental and the estimated equilibrium values obtained from the isotherm models, error functions such as the APE (Average percent errors) (Eq. (10)), *X*^2^ (Chi-square error) (Eq. (11)), HYBRID (Hybrid error function) (Eq. (12)), MPSD (Marquardt’s Percent standard deviation) (Eq. (13)), EABS (Sum of absolute errors) (Eq. (14)), and RMS (Root mean square errors) (Eq. (15)) are the primary error functions used^74^. As shown in Table 2, the error investigation expressing the similarity between the experimental data from the MBT and the values calculated using the theoretical isotherms is made and it becomes evident that the LIM is the best appropriate isotherm model since it has the lowest error function values APE, X2, RMS, HYBRID, EABS, and EABS and the lowest error function values (APE), (X2), and (RMS) (MPSD). As a result, the Langmuir isotherm model is the most appropriate in terms of *R*^2^, it is obvious that when models are compared, the Langmuir isotherm model has the lowest calculated values of the error functions.

**Table 2:** In the adsorption of AY11 dye by MBT, a few error function values of the isotherm models were found to be the most closely matched to the experimental equilibrium data.

Isotherm model	APE (%)	X^2^	Hybrid	MPSD	EABS	RMS
LIM	0.008	0.019	0.082	0.039	10.027	0.038
FIM	0.030	0.284	1.234	0.155	38.228	0.148
TIM	0.024	0.186	0.810	0.123	31.501	0.118
DRIM	47.159	1470.28	50.527	74.806	1628.64	72.270

10 $$APE\left(\%\right)=\frac{100}{N}\sum_{i=1}^{N}{\left|\frac{{q}_{e,isotherm}-{q}_{e,calc}}{{q}_{e,isotherm}}\right|}_{i},$$

11 $${X}^{2}=\sum_{i=1}^{N}\frac{{\left({q}_{e,isotherm}-{q}_{e,calc}\right)}^{2}}{{q}_{e,isotherm}},$$

12 $$HYBRID=\frac{100}{N-P}\sum_{i=1}^{N}{\left|\frac{{q}_{e,isotherm}-{q}_{e,calc}}{{q}_{e,isotherm}}\right|}_{i},$$

13 $$MPSD=100\sqrt{\frac{1}{N-P} \sum_{i=1}^{N}(\frac{{q}_{e,calc}-{q}_{e,isotherm}}{{q}_{e,isotherm}}{)}_{i}^{2}},$$

14 $$EABS=\sum_{i=1}^{N}{\left|{q}_{e,calc}-{q}_{e,isotherm}\right|}_{i},$$

15 $$RMS=100\sqrt{\frac{1}{N}\sum_{i=1}^{N}(1- \frac{{q}_{e,calc}}{{q}_{e,isotherm}}{)}^{2}.}$$

### Adsorption kinetic studies

**Figure 11:**
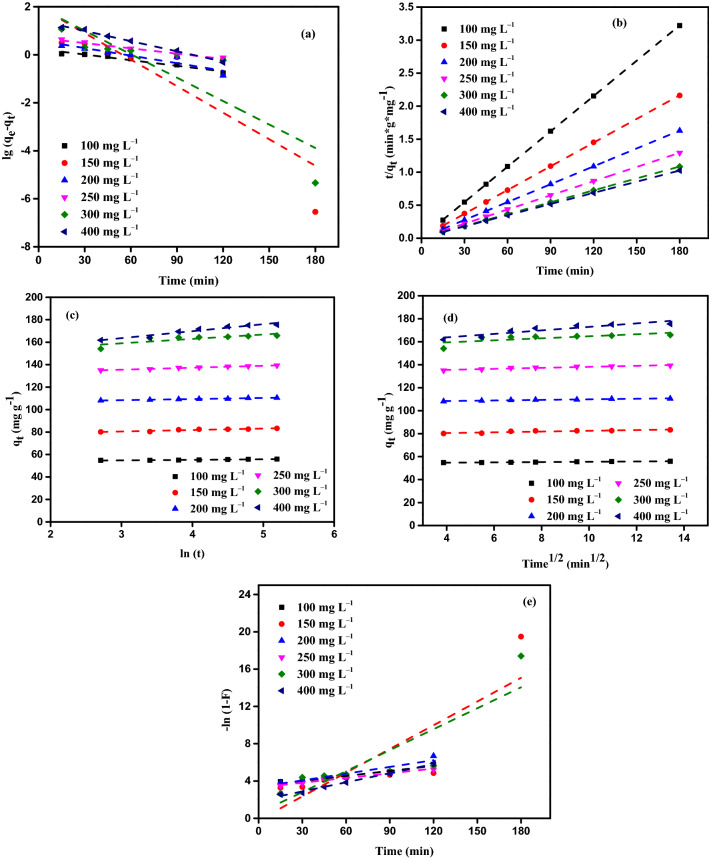
(**a**) The PFO, (**b**) The PSO, (**c**) The EKM, (**d**) The IPD model (**e**), and The FD model curves of the AY11 dye removal by MBT [AY11 dye starting concentration (100–400 mg L^–1^), MBT dose (1.75 g L^−1^), RT (24 ± 2 °C).

The PFO (pseudo-first-order) (Eq. (16)), the PSO (pseudo-second-order) (Eq. (17)), the EKM (Eq. (18)), the IPD (Eq. (19)), and the Film diffusion (Eq. (20)) equations were all used in the development of the kinetic studies of AY11 dye adsorption on MBT^74^. Generally, the correlation coefficients (*R*^2^) of the kinetic equations shown in Tables 3, 4 and 5 have values ranging from 0 to 1, and the applicability of the equations as a suitable model is inversely proportional to how near the *R*^2^ value is to one (1). Using the linear plot of log(*q*_e_–*q*_t_) values with time (*t*), as presented in Fig. 11a, the *k*_1_ (rate constant) and *q*_e_ may be determined from the rate constant (*k*_1_).

16 $${\text{log}}\left({q}_{e}-{q}_{t}\right)={\text{log}}{q}_{e}-\frac{{K}_{1}}{2.303}t,$$

17 $$\frac{t}{{q}_{t}}=\frac{1}{{K}_{2}{q}_{e}^{2}}+\frac{t}{{q}_{e}},$$

18 $${q}_{t}=\frac{1}{\beta }{\text{ln}}\left(\propto \beta \right)+\frac{1}{\beta }{\text{ln}}\left(t\right),$$

19 $${q}_{t}={K}_{dif}{t}^{0.5}+C,$$

20 $${\text{ln}}\left(1-F\right)={K}_{FD}\left(t\right),$$

where *K*_FD_ *and F* (*F* = *q*_*t*_/*q*_*e*_) are the film diffusion rate constant, and the fractional attainment of equilibrium, respectively. The low *R*^2^ values are associated with a considerable variation between the computed and the experimental values of *q*_e_, and vice versa. When taking into consideration the findings in Table 3, it can be concluded that the kinetic equation of the PFO is not particularly suitable for the removal of AY11 dye by MBT. When the MBT concentration is increased from 0.75 to 1.75 g L^–1^, as reported in Table 3, there is no regular increase or reduction in the values of the correlation coefficient (*R*^2^).

**Table 3:** The findings of the AY11 dye removal by MBT adsorbent using the PFO and the PSO parameters.

Parameter			PFO			PSO			
MBT (g L^−1^)	AY11 dye (mg L^–1^)	*q*_*e*_ (exp.)	*q*_*e*_ (calc.)	*k*_1_	R^2^	*q*_*e*_ (calc.)	*k*_2_ × 10^3^	*h*	R^2^
0.75	100	126.98	7.9	15.20	0.682	129.9	4.20	70,922	1.000
	150	192.12	137.2	30.63	0.939	200.0	0.97	38,759.7	1.000
	200	254.36	413.1	70.47	0.852	277.8	0.50	38,759.7	0.999
	250	319.18	3383.0	72.31	0.739	357.1	0.10	12,437.8	0.986
	300	329.21	6105.2	83.83	0.677	344.8	0.12	14,771.0	0.983
	400	374.86	2860.9	69.55	0.733	400.0	0.13	21,008.4	0.990
1.00	100	95.72	24.0	49.74	0.759	97.1	9.91	93,457.9	1.000
	150	144.21	20.9	21.42	0.797	147.1	1.92	41,493.8	1.000
	200	192.28	414.4	77.84	0.846	196.1	1.38	52,910.1	1.000
	250	240.77	2897.3	97.65	0.777	250.0	0.52	32,258.1	1.000
	300	283.17	116.5	30.17	0.789	256.4	0.43	28,409.1	0.999
	400	320.24	142.7	25.10	0.945	303.0	0.35	32,362.5	0.998
1.25	100	76.96	2.2	6.91	0.539	78.1	12.80	78,125.0	1.000
	150	115.56	140.1	73.93	0.784	116.3	4.68	63,291.1	1.000
	200	154.35	17.2	31.32	0.924	156.3	4.31	105,263.2	1.000
	250	193.05	28.2	20.50	0.903	196.1	1.45	55,555.6	1.000
	300	232.08	41.4	24.18	0.808	204.1	1.10	45,662.1	1.000
	400	304.51	105.1	26.71	0.929	250.0	0.48	29,761.9	0.999
1.50	100	64.33	37.6	74.39	0.721	65.4	20.36	86,956.5	1.000
	150	97.10	6.9	19.58	0.993	98.0	6.98	67,114.1	1.000
	200	130.00	114.3	77.61	0.752	129.9	7.32	123,456.8	1.000
	250	162.60	5.0	19.58	0.986	161.3	9.86	256,410.3	1.000
	300	194.13	455.4	80.61	0.845	172.4	1.59	47,393.4	1.000
	400	258.78	47.1	21.65	0.952	208.3	0.95	41,152.3	0.999
1.75	100	55.45	1.1	17.96	0.960	56.2	25.55	80,645.2	1.000
	150	83.51	1.8	87.51	0.305	83.3	12.63	87,719.3	1.000
	200	111.67	3.5	9.67	0.722	111.1	15.00	185,185.2	1.000
	250	139.79	102.0	84.98	0.706	138.9	8.94	172,413.8	1.000
	300	167.74	95.1	75.08	0.760	166.7	7.06	196,078.4	1.000
	400	224.64	26.2	32.70	0.993	178.6	2.85	90,909.1	1.000

**Table 4:** Results of removal of AY11 dye by MBT using EKM, IPD, and FD model parameters.

Parameter		EKM			IPD			FD	
MBT (g·L^−1^)	AY11 dye (mg L^–1^)	*α*	*β*	*R*^2^	*К*_*dif*_	*C*	*R*^2^	*К*_*FD*_	*R*^2^
0.75	100	9 × 10^7^	0.16	0.723	1.384	113.420	0.565	0.026	0.860
	150	2041.9	0.05	0.823	4.771	138.480	0.668	0.033	0.980
	200	82.95	0.02	0.918	11.388	126.350	0.789	0.040	0.991
	250	91.42	0.02	0.934	16.813	97.433	0.979	0.018	0.938
	300	75.99	0.02	0.951	14.508	122.900	0.985	0.010	0.980
	400	154.06	0.02	0.949	15.584	169.810	0.982	0.017	0.906
1.00	100	11 × 10^23^	0.62	0.960	0.399	92.006	0.880	0.014	0.905
	150	48,360	0.09	0.853	2.572	115.480	0.704	0.034	0.967
	200	12,065	0.06	0.783	3.914	148.840	0.621	0.026	0.923
	250	451.38	0.03	0.866	7.525	151.830	0.727	0.031	0.098
	300	659.06	0.03	0.950	7.430	154.770	0.864	0.041	0.079
	400	722.92	0.03	0.950	8.832	180.840	0.880	0.018	0.963
1.25	100	6 × 10^25^	0.83	0.929	0.298	74.126	0.849	0.014	0.876
	150	6 × 10^12^	0.29	0.945	0.870	105.350	0.882	0.025	0.958
	200	1.7 × 10^12^	0.20	0.867	1.156	140.560	0.726	0.031	0.924
	250	1.6 × 10^5^	0.07	0.887	3.188	154.520	0.756	0.026	0.909
	300	25,082	0.06	0.841	3.892	152.340	0.734	0.024	0.808
	400	4255	0.04	0.919	6.072	166.210	0.736	0.027	0.929
1.50	100	1.9 × 10^41^	1.55	0.980	0.167	62.929	0.994	0.016	0.974
	150	5.5 × 10^16^	0.44	0.989	0.563	89.912	0.983	0.020	0.993
	200	2.9 × 10^24^	0.47	0.991	0.530	122.060	0.885	0.021	0.972
	250	1.2 × 10^39^	0.59	0.978	0.417	154.560	0.948	0.020	0.986
	300	1.1 × 10^7^	0.11	0.968	2.236	141.650	0.500	0.039	0.852
	400	1.1 × 10^6^	0.08	0.932	3.179	162.740	0.565	0.022	0.952
1.75	100	1.3 × 10^47^	2.05	0.933	0.129	54.234	0.974	0.018	0.960
	150	2.3 × 10^25^	0.76	0.889	0.327	82.287	0.821	0.016	0.803
	200	1.9 × 10^46^	1.01	0.977	0.251	110.740	0.943	0.024	0.878
	250	3.5 × 10^32^	0.57	0.987	0.440	135.440	0.934	0.017	0.974
	300	7.9 × 10^16^	0.26	0.667	0.887	161.520	0.512	0.022	0.750
	400	9.0 × 10^10^	0.16	0.937	1.527	195.350	0.855	0.033	0.993

**Table 5:** Reported the *Q*_m_ of different azo dyes by various bio-materials.

Materials	Azo dye	*Q*_m_ (mg g^−1^)	Removal (%)	Refs.
MBT	Acid Yellow 11	384.62	96.76	This work
Peanut husk-ethylenediamine	Sunset Yellow	117.70	–	^35^
Shells of Mandarin	Basic Blue 9	294.00	–	^58^
	Acid Yellow 36	417.00	–	
Shells of macore fruit	MO dye	3.42	82.73 (MO)	^68^
	Methylene Blue (MB)	10.61	91.31 (MB)	
Carbon of OP	Direct Yellow 12	75.76	98.10	^73^
Biochar of rice husk-sludge	Direct Red	59.77	–	^79^
	Acid Orange II	42.12	–	
	React Blue 19	38.46	–	
	MB dye	22.59	–	
Biochar of wood wastes	Indosol Black NF1200	185.00	99.00	^80^
Biochar of MP	MO dye	16.27	99.00 (MO)	^81^
	Fast Green (FG)	12.44	99.00 (FG)	
Carbon of MP	MB dye	313.00	–	^82^
	Metanil Yellow	455.00	–	
*N*-doped biochars from phragmites Australis	Acid Red 18	134.17	–	^83^
Carbonized MP	MB dye	196.08	99.77 (MB)	^84^
	MO dye	–	79.87 (MO)

The PSO kinetic equation was also applied to study the adsorption of AY11 dye by MBT, which was found to be effective. In Fig. 11b, it is possible to determine *k*_2_ (g mg^–1^ min^–1^) (kinetic constant) of the PSO and the equilibrium adsorbed quantity of AY11 dye (*q*_e_) by graphing the ratio of time to amount of dye adsorbed (*t*/*q*_e_) over time. MBT adsorption with dye (AY11) is depicted in Fig. 11b, which shows the PSO kinetic plot of MBT. In addition, the values of the kinetic constant (*k*_2_), the values of *q*_e_ that have been experimentally and theoretically determined, and the corresponding values of the *R*^2^ of the PSO equation are also included in Table 3. The PSO model is the one that has *R*^2^ values that are the closest to 1. In order to account for this, the pseudo-second-order model is the most appropriate kinetic model. As a result, for all starting AY11 dye concentrations investigated, the *q*_e_ values computed by the PSO equation and the experimental values of *q*_e_ are exactly the same as each other.

The EKM is another kinetic equation that was determined in the adsorption of AY11 dye using MBT, and Fig. 11c depicts the correlation plot of *q*_t_ and ln (*t*) of the EKM. It was decided to use the intercept and slope from Fig. 11c for the computation of EKM's constants and the results provided in Table 4. Using the *R*^2^ values as a benchmark, it can be asserted that the *R*^2^ values of the EKM equation are higher than the values of the PFO equation and lower than the values of the PSO equation (when the values of *R*^2^ of the different models are compared) (Tables 3, 4). Following the findings in Tables 3 and 4, it is clear that in some circumstances, chemical adsorption can influence the removal rate of AY11 dye by MBT.

Solid–liquid adsorption is explained by applying the IPD equation, which is employed to explain solute transfer. It is possible to identify and explain all of the steps in the sorption process using the IPD model, which is described below. In a process of adsorption, the adsorbate is deposited onto the adsorbent in 3 processes that are carried out in succession: (i) The first stage involves the ions or molecules’ movement carried from the solution through the liquid film to the adsorbent surface. (ii) The second step is the diffusion of the ions or molecules that have been contacted to the surface of the adsorbent. (iii) A chemical reaction occurs with the active groups on the surface of the adsorbent, which is the final stage in the removal process. There is a distinct time lag between each of these three phases, and the one that takes the longest to complete is also the rate-determining step of the adsorption.

The IPD step should be used to regulate the adsorption, according to the principle of Ref.^78^, if the lines drawn in the graphs of *q*_t_ and root time (*t*^0.5^) in Fig. 11d pass through the origin, it is advised that this step be used to control the adsorption. It is considered, on the other hand, that in the scenario when the drawn line lines do not pass through the origin (i.e., when the *C* value is large), the rate of the removal process is dictated by the rate of the film diffusion (FD). As shown in Fig. 11d, the Webber–Morris adsorption line for the removal of the AY11 dye using MBT at varied initial AY11 dye concentrations and varying adsorbent dosages is depicted. Measured from the slope and intercept of the plot of *q*_t_ vs *t*^0.5^, respectively, the values of *K*_dif_ and *C* were mentioned in Table 4. Figure 11d shows that due to the high *C* intersection of the straight lines representing all adsorbent concentrations, they do not pass through the origin. There are several possible explanations for this condition, one of which is that the removal rate of the AY11 dye using MBT grows continuously with time and that this rate is controlled by the FD model (Fig. 11e). The reduction in the surface area and volume of the pore of the MBT that occurs throughout the process of adsorption is the cause of the FD process.

### The optained results compared to those found in the literature

The effectiveness of azo dye elimination using variable adsorbents summarised in the literature was compared to the MBT adsorbent (Table 5), because there has been no investigation into the elimination of Acid Yellow 11 dye. This demonstrated that MBT was effective for the adsorption of AY11 dye. The MBT shows *Q*_m_ (384.62) and removal percentage (96.76%) which are comparable to those reported in Table 5 for various adsorbent for removal of different dyes.

## Conclusion

In this work, it was demonstrated that MP, which is agricultural waste, can be applied to generate an economical and effective adsorption material that is also environmentally friendly. In order to make Mandarin-Biochar-TETA (MBT) for use in the adsorption of AY11 dye, an azo dye, the dried mandarin peel was treated with 80% H_2_SO_4_ at 200 °C, then oxidised with ozone, and finally aminated by reaction with triethylenetetramine (TETA). It is discovered that the intake of AY11 dye is dependent on the adsorbent dose, the starting concentration, the contact duration, and the value of the pH of the aqueous solution. Using MBT as a model, it was discovered that the ideal pH for AY11 dye adsorption by MBT was 1.5. When the 1.75 g L^–1^ of MBT dosage was employed, it was discovered that the higher elimination of the AY11 dye and the *q*_e_ were both determined to be in equilibrium with each other. The Langmuir model outperforms all other sorption models when it comes to removing AY11 dye from water. Because the LIM provided a better description of the sorption of AY11 dye onto MBT, the *Q*_m_ computed using the LIM was measured to be 384.62 mg g^–1^ for AY11 dye. When the adsorption energy values from DRIM were taken into consideration, it was established that chemical adsorption had occurred in this case. This makes it conceivable to use Mandarin-Biochar-TETA (MBT) as a viable adsorbent for the adsorption of AY11 dye from aquatic environments, which has the added benefit of being environmentally friendly.

## Data Availability

The raw data used and/or analysed during the current study available from the corresponding author on reasonable request.
